# Growth-regulated co-occupancy of Mediator and Lsm3 at intronic ribosomal protein genes

**DOI:** 10.1093/nar/gkae266

**Published:** 2024-04-13

**Authors:** Wael R Abdel-Fattah, Mattias Carlsson, Guo-Zhen Hu, Ajeet Singh, Alexander Vergara, Rameen Aslam, Hans Ronne, Stefan Björklund

**Affiliations:** Department of Medical Biochemistry and Biophysics, Umeå University, SE-901 87 Umeå, Sweden; Department of Forest Mycology and Plant Pathology, Swedish University of Agricultural Sciences, Box 7026, SE-750 07 Uppsala, Sweden; Department of Forest Mycology and Plant Pathology, Swedish University of Agricultural Sciences, Box 7026, SE-750 07 Uppsala, Sweden; Department of Medical Biochemistry and Biophysics, Umeå University, SE-901 87 Umeå, Sweden; Department of Plant Physiology, Umeå University, SE-901 87 Umeå, Sweden; Department of Medical Biochemistry and Biophysics, Umeå University, SE-901 87 Umeå, Sweden; Department of Forest Mycology and Plant Pathology, Swedish University of Agricultural Sciences, Box 7026, SE-750 07 Uppsala, Sweden; Department of Medical Biochemistry and Biophysics, Umeå University, SE-901 87 Umeå, Sweden

## Abstract

Mediator is a well-known transcriptional co-regulator and serves as an adaptor between gene-specific regulatory proteins and RNA polymerase II. Studies on the chromatin-bound form of Mediator revealed interactions with additional protein complexes involved in various transcription-related processes, such as the Lsm2–8 complex that is part of the spliceosomal U6 small nuclear ribonucleoprotein complex. Here, we employ Chromatin Immunoprecipitation sequencing (ChIP-seq) of chromatin associated with the Lsm3 protein and the Med1 or Med15 Mediator subunits. We identify 86 genes co-occupied by both Lsm3 and Mediator, of which 73 were intron-containing ribosomal protein genes. In logarithmically growing cells, Mediator primarily binds to their promoter regions but also shows a second, less pronounced occupancy at their 3′-exons. During the late exponential phase, we observe a near-complete transition of Mediator from these promoters to a position in their 3′-ends, overlapping the Lsm3 binding sites ∼250 bp downstream of their last intron–exon boundaries. Using an unbiased RNA sequencing approach, we show that transition of Mediator from promoters to the last exon of these genes correlates to reduction of both their messenger RNA levels and splicing ratios, indicating that the Mediator and Lsm complexes cooperate to control growth-regulated expression of intron-containing ribosomal protein genes at the levels of transcription and splicing.

## Graphical Abstract

**Graphical Abstract:**
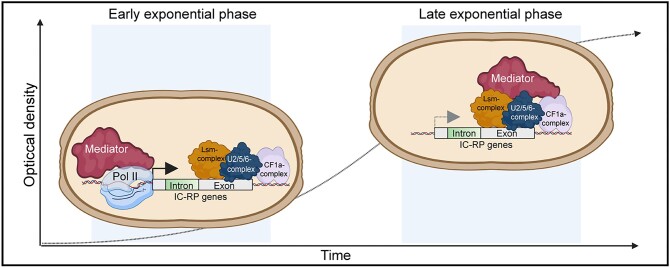

## Introduction

Mediator is an essential transcriptional co-regulator transmitting signals from promoter-bound activators and repressors to the RNA polymerase II (Pol II) preinitiation complex (1–3), and it is also involved in transcription termination of some genes (4). We previously used ChIP-seq to identify yeast Mediator occupancy at gene promoters but also at multiple, nonpromoter chromatin regions, so-called chromosomal interaction domain boundaries (5). These experiments were combined with biochemical methods to show that Mediator isolated from the chromatin fraction of the yeast *Saccharomyces cerevisiae*, in contrast to traditionally purified Mediator from the soluble (nonchromatin) fraction, interacts with an additional set of 88 proteins. Thirty-eight of these proteins were found to be subunits of protein complexes involved in different activities related to transcription, for example chromatin remodeling [RSC (6)], messenger RNA (mRNA) 3′-end processing [CPF and CF1A (7)] and gene looping [Ssu72 (8)]. We also identified Mediator-associated protein complexes that are mostly known for their cytoplasmic functions, but have also been found to shuttle in and out of the nucleus. Examples of such complexes are Arp2/3, involved in actin nucleation (9), and the Lsm complex that is engaged in mRNA processing activities such as nuclear pre-mRNA splicing and cytoplasmic mRNA decay (10). The chromatin locations where Mediator interacts with these proteins and complexes have so far not been identified.

Lsm constitutes a family of RNA binding proteins found in virtually all organisms from prokaryotes to eukaryotes (11). They assemble into rings of six to seven Lsm protein molecules and play various roles in mRNA processing and regulation. *Saccharomyces cerevisiae* encodes nine Lsm proteins and most of them are essential for growth (12). In most reports, the Lsm1–7 complex is described to be cytoplasmic where it promotes mRNA decapping and decay (13). In contrast, the Lsm2–8 complex is considered to have a nuclear localization and to function in splicing (14). However, also a complex of Lsm1–7, Pat1 and additional proteins has been shown to be present in the nucleus and to interact with gene promoters (15). In our previous study of interactors with the chromatin-bound form of Mediator (5), we identified all subunits of the Lsm1–7/Pat1 complex. However, we did not detect any Lsm8. Structure-guided mutagenesis of genes encoding subunits of the nuclear Lsm2–8 ring showed that the lethality of deletions of these genes can be suppressed by overexpression of U6 small nuclear RNA (snRNA) or the U6 small nuclear ribonucleoprotein subunit Prp24, indicating that stabilization of U6 snRNA is an essential function of the yeast Lsm2–8 proteins (16). Here, we present results from ChIP-seq experiments identifying chromatin regions and genes that are co-occupied by Lsm3 and Mediator globally, and RNA sequencing (RNA-seq) experiments describing the functional impact of Mediator–Lsm interactions on transcription and splicing of ribosomal, intron-containing (IC) genes in *S. cerevisiae*.

## Materials and methods

### Yeast strains and growth conditions

The wild-type haploid *S. cerevisiae* strain BY4742 (*MAT***a** his3Δ1 leu2Δ0 met15Δ0 ura3Δ0) was used both for ChIP-seq of Mediator subunits Med1 and Med15 and for purification of RNA for RNA-seq experiments. Derivatives of BY4742 containing C-terminally tandem affinity purification (TAP)-tagged versions of Lsm3 protein (Horizon) or Rna14 protein (Open Biosystems) were also used in the ChIP-seq experiments. For experiments using cells at different growth phases, single colonies of *S. cerevisiae* BY4742 cells were inoculated into 10 ml of YPD medium (1% yeast extract, 2% bacto-peptone and 2% glucose), precultivated in YPD at 30°C, diluted in fresh YPD to an OD_600nm_ of 0.1–0.15 and cultivated for 2 h at 30°C, after which the first samples (labeled 0 h in the figures) were harvested. Subsequent samples were harvested at 2, 4 and 6 h after the 0 h point for ChIP-seq or RNA-seq assays.

### Purification of Med1 and Med15 antibodies

Immunoglobulins were purified using Protein A-Sepharose from sera of rabbits (Agri Sera AB) immunized with a mixture of two synthetic peptides (C^4^APVQDKDTLSNAER^17^ and C^1049^EKQEVTNEAPFLTS^1062^ of Med15) derived from the Med15 protein, or with a full-length purified recombinant Med1 protein expressed from the pET6xhisMed1 plasmid in *Escherichia coli* strain BL21-DE3 as previously described (17,18). Specific antibodies against Med1 or Med15 subunits of Mediator were affinity purified using Med1-coupled or Med15 peptide mixture-coupled Dynabeads, respectively.

### Co-immunoprecipitation of Med1 and Lsm3 from the chromatin fraction of cell lysates

Cells of an *LSM3-TAP* strain (Open Biosystems) were grown at 30°C in 50–60 l of YPD medium to an OD_600nm_ of 4. Cell lysis and separation of chromatin fractions were performed using the standard protocol (19), except that we used ammonium sulfate to 0.5 M final concentration instead of 1 M to release chromatin-associated proteins, as we have described previously (5). For immunoprecipitation, 100 μl of Protein A/G-coupled magnetic beads (Thermo Scientific) were incubated with 75 μl of α-Protein A antibody (Sigma, #P3775-1VL) at 4°C for 1 h. Aliquots of 250 μl of chromatin extracts were added to the beads and further incubated at 4°C on a rotator overnight. The next day, flow-through was removed and the beads were washed three times with 500 μl buffer X [40 mM HEPES, pH 7.6, 10% glycerol, 150 mM KOAc, 1 mM dithiothreitol with protease inhibitors (Roche Applied Science) and phosphatase inhibitors (Roche Applied Science)]. The washes were pooled and precipitated with 15% trichloroacetic acid (TCA; Sigma). Beads and TCA-precipitated proteins in the wash were incubated in 30 μl of sodium dodecyl sulfate (SDS) loading dye followed by heating at 95°C for 5 min. The input sample was diluted 10 times before loading on the gel. Samples from the immunoprecipitation were separated by 4–15% (Bis-Tris) sodium dodecyl sulfate–polyacrylamide gel electrophoresis (SDS–PAGE; Bio-Rad) and proteins were transferred to polyvinylidene fluoride (PVDF) membranes by blotting. The membrane was cut into two pieces based on the molecular weight of the proteins and probed with α-Protein A or α-Med1 antibodies followed by incubation with horseradish peroxidase (HRP) conjugating Clean-Blot™ detection reagent (Thermo Scientific, #21230) and visualization using the ChemiDoc gel documentation system (Bio-Rad).

### ChIP-seq

ChIP-seq was essentially performed as previously described (20) with the following modifications. Cells were subjected to cross-linking (0.8% formaldehyde) for 15 min at room temperature. Cross-linking was stopped by the addition of 125 mM glycine to the cultures. Cells were collected and lysed in lysis/IP buffer [140 mM NaCl, 1 mM ethylenediaminetetraacetic acid (EDTA), 1% Triton X-100, 0.1% sodium deoxycholate, 50 mM HEPES/KOH, pH 7.5, 1 mM phenylmethylsulfonyl fluoride (PMSF) and protease inhibitors (Roche Applied Science)]. Lysis was performed at 4°C using 0.5 mm glass beads (BioSpec Products, Inc.) in a FastPrep-24 machine (5 × 1-min bursts at a speed of 6.0 m/s). To obtain sheared chromatin fragments between 200 and 500 bp in size, we used a Covaris^®^ E220 focused ultrasonicator for 12 min (settings: peak intensity power of 150 W with a duty factor of 28%, 200 cycles per burst at 3–7°C). Sonicated chromatin was incubated with 10–20 μg of IgG-coupled magnetic beads (Novex^®^ from Life Technologies) to detect Lsm3-TAP and Rna14-TAP, or with anti-Med1 or anti-Med15 antibodies bound to Protein A-coupled magnetic beads to detect Med1 and Med15. Following overnight rotation at a speed of 10 rpm at 4°C, the magnetic beads were washed three times with lysis/IP buffer and once with TE buffer (10 mM Tris, pH 8.0, 1 mM EDTA). After washing, bound DNA was eluted at 65°C in elution buffer (TE buffer containing 1% SDS) for 10 min and incubated at 65°C overnight to reverse cross-links. After the reversal of cross-linking, immunoprecipitated DNA was treated sequentially with RNase A and proteinase K to digest RNA and proteins. The DNA fragments were then cleaned using the ChIP DNA Clean & Concentrator™ kit (Zymo Research). Aliquots of 1–2 ng of DNA from each sample were tested for proper shearing using a fragment analyzer (Agilent 2100 Bioanalyzer G2938C), and then sent to SciLife National Genomics Infrastructure (Sweden) for library preparation using the SMARTer ThruPLEX kit and subsequent paired-end sequencing using Illumina NovaSeq S4-200 cycle flow cells in individual lanes.

### Processing of ChIP-seq Illumina sequencing data

On average, each Lsm3-TAP ChIP-seq experiment produced 5–6 million mapped reads from Bowtie2 paired-end alignment to the sacCer3 reference genome. After mapping of reads, we called narrow peaks at an average fragment size of 300 bp and a false discovery rate of <0.05. ChIP-seq using an untagged wild-type strain was used as control for peak calling and enrichment analyses. We obtained positive linear correlation profiles between three biological replicates on the entire genome using a bin size of 1 kb (Supplementary Figure S1A and B). Next, we filtered out peaks that were detected in all three replicates and showed >2-fold enrichment in at least one of the replicates, to identify chromatin regions that were significantly enriched for Lsm3 occupancy.

For Mediator ChIP-seq experiments, we used Dynabeads-coupled, affinity-purified antibodies specific for the Med1 (middle module) or Med15 (tail module) subunit with samples from the wild-type *BY4742* strain grown in YPD that were collected at different time points during the exponential growth phase. Alignments and peak callings were performed as described for the Lsm3-TAP ChIP (see above). Positive linear correlation profiles were obtained between three biological replicates of each ChIP-seq on the entire genome using a bin size of 1 kb (Supplementary Figure S1C and D). We identified genomic regions of Mediator occupancy (>2-fold above background) genome-wide. The genomic regions (±500 bp) enriched in Mediator ChIP-seq were used to retrieve lists of Med1 and Med15 co-occupied genes using the YeastMine database. We manually removed a set of genes that were located close to broad diffuse peaks, genes where the peaks were found downstream from the coding sequences (CDS) and genes where the peaks overlapped with hyper-ChIPable regions (HCRs), dubious open reading frames (ORFs), noncoding RNA genes, autonomously replicating sequences, centromeres or transposable elements using the Integrative Genomics Viewer (IGV). For comparison and validation, we used score files from previously published ChIP-seq data (5) for a set of TAP-tagged Mediator subunit strains representing each of the four Mediator modules: head (TAP-Med17), middle (Med1, TAP-Med14, TAP-Med19), tail (TAP-Med3, Med15, TAP-Med15) and kinase (TAP-CycC). The ChIP-seq data using Med1 and Med15 antibodies were combined with the data using TAP-tagged strains and the co-occupied genes were arranged into four distinct groups by *k*-means clustering.

### ChIP-qPCR

To validate the ChIP-seq results and to study occupancy for each of the Lsm1, Lsm3 and Lsm8 proteins, we employed reverse transcription quantitative polymerase chain reaction (RT-qPCR) utilizing extracts isolated from yeast strains expressing each of these proteins with 3× Flag-epitope tags. Cells were processed as described for the ChIP-seq experiments above to obtain DNA fragments for ChIP-qPCR. Sonicated chromatin was incubated with 10–20 μg of anti-rabbit IgG magnetic Dynabeads (Invitrogen) or anti-FLAG M2 magnetic beads (Sigma–Aldrich) at 4°C with rotation overnight. The beads were washed twice with 500 μl cold IP buffer [140 mM HEPES–KOH, pH 7.5, 150 mM NaCl, 1 mM EDTA, 1% Triton X-100, 0.1% sodium deoxycholate, 1 mM PMSF and Roche cOmplete™ Protease Inhibitor Cocktail (Sigma–Aldrich)] and once with cold Tris–EDTA buffer (10 mM of Tris–HCl, pH 8.0, 1 mM EDTA). The cross-linked DNA–protein complexes were eluted by incubation with 150 μl elution buffer (Tris–EDTA buffer + 1% SDS) at 65°C for 10 min. Cross-links were reversed by incubation overnight at 65°C. Proteinase K was added to a final concentration of 0.67 mg/ml and the samples were incubated at 42°C for 2–4 h. DNA was isolated using phenol/chloroform extraction and ethanol precipitation. The input as well as the IgG and Flag-immunoprecipitated DNA samples were analyzed by qPCR using primers specific for *RPL19B* (5′-GGAATCCAAGGGTAACGCCT-3′ and 5′-CAGCTTCTTCGTTCAAAGCC-3′), *RPL23A* (5′-CCAGCTATTGTTGTCCGTCA-3′ and 5′-CCCTTAGGATTAGCGATGACAC-3′), *snR17B* (5′-AATGGCGCGATGATCTTGAC-3′ and 5′-GTCAGACTGCCATTTGTACCC-3′) and *RPL29* (5′-GGCTCACAGAAACGGTATCA-3′ and 5′-CCTTAGCAGTGCCGTGTAG-3′).

### RNA-seq and processing of Illumina sequencing data

*Saccharomyces cerevisiae* BY4742 cells were grown as described above. We used 24 biological replicates for the 0 h point and 6 biological replicates for each subsequent time point. Total RNA was prepared from each sample with the RNeasy Mini Kit (Qiagen) using the protocol of the kit manufacturer for purification of total RNA from yeast by enzymatic lysis. Sequencing was performed by the SNP&SEQ Technology Platform, Science for Life Laboratory, Uppsala, Sweden. Sequencing libraries, including polyA selection, were prepared from 500 ng of total RNA using the TruSeq stranded mRNA library preparation kit (Illumina, Inc.). Further quality control and generation of counts of reads mapped to genes using both StringTie and STAR aligners in combination with featureCounts as part the nfcore/rnaseq best-practice analysis pipeline was performed by the National Genomics Infrastructure, Uppsala, Sweden (https://github.com/nf-core/rnaseq). The ComBat-seq program was used to adjust the data for batch differences in the efficiency of polyA selection. We regenerated the counts of mapped reads using featureCounts with two different settings, counting either over the entire genes or over exons only. A total of 376 IC genes from the *Saccharomyces* Genome Database (SGD) were filtered for the presence of introns with a minimum intron length of 25 bp according to the Saccharomyces_cerevisiae.R64-1-1.106.gtf genome annotation. We used a constraint of a minimum of 75 mapped reads while allowing for zero mapped reads within the introns of each IC gene. Finally, we removed genes associated with HCRs. This filtering left 141 IC genes that were used for further analysis. We calculated intron mapped read counts as the difference between whole gene counts and exon-only counts and estimated the fraction of splicing in each IC RNA species by considering the relative length of introns and exons, obtaining a splice ratio value between 0 (no splicing) and 1 (fully spliced). Counts of reads mapped to genes (gene counts) were used when analyzing and comparing expression levels after normalization by scaling each sample library to the average library size. Data points of splice ratios and gene counts in figures were taken as averages of samples at each time point or over all time points.

## Results

### Lsm3 interacts with chromatin-associated Mediator

**Figure 1.**
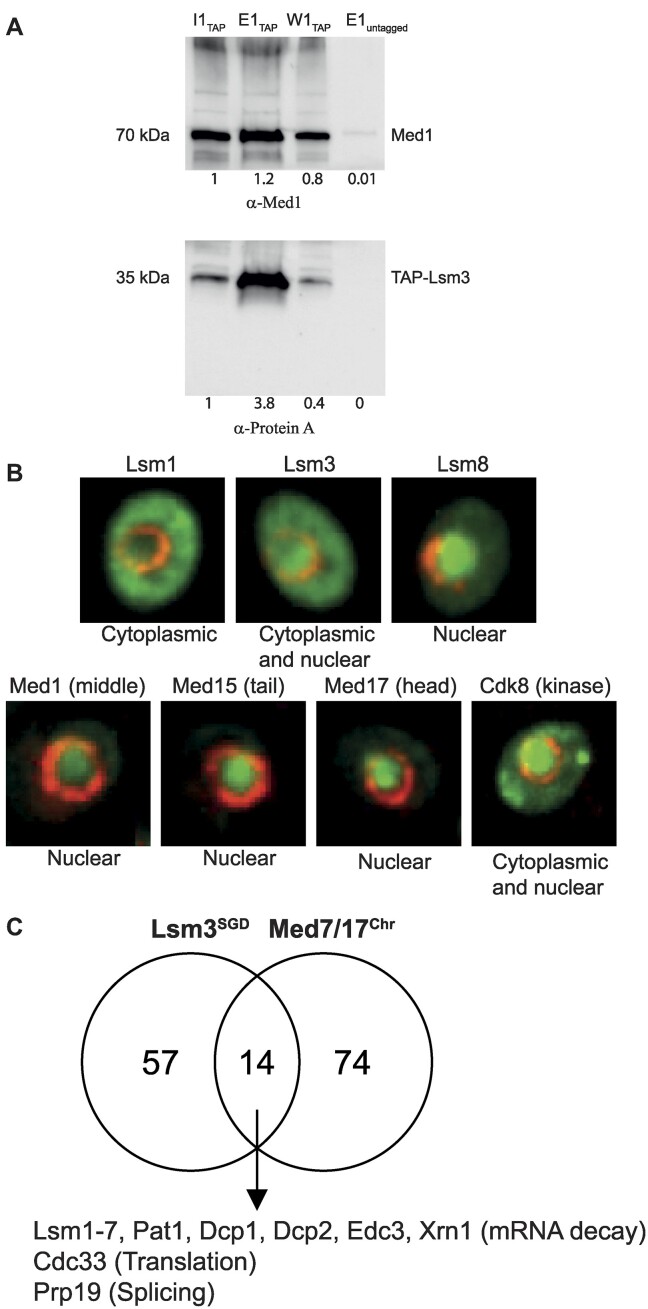
Lsm3 chromatin association and interaction with Mediator. (**A**) Co-immunoprecipitation of Med1 and Lsm3-TAP from chromatin. Chromatin extract was isolated from a strain expressing Lsm3-TAP and immunoprecipitated using IgA magnetic beads. Beads were washed with buffer A and proteins bound to the beads were eluted using 1× SDS sample buffer. Proteins from input (I, 10 times diluted), eluate (E) and wash (W) were resolved on 4–15% SDS–PAGE, transferred to a PVDF membrane and blotted using α-Protein A (lower part) or α-Med1 (upper part) antibodies followed by incubation in Clean-Blot™ IP (HRP) detection reagent (Thermo Scientific). Quantifications of bands were conducted using the ImageJ software (https://imagej.net/ij/) and normalized to the input (set to 1). (**B**) Cellular localization of Lsm and Mediator protein subunits. Selected images representing the indicated proteins were retrieved from the YeastRGB open-source initiative (http://www.yeastrgb.org/). The green fluorescence is from each ORF under the control of their native promoters and C-terminally fused to mNeonGreen followed by their endogenous terminator. The red fluorescence is from expressing NUP49 tagged with mCherry to highlight the nuclear membrane. (**C**) Venn diagrams representing the overlap between the 88 proteins previously shown to interact with Med7 and Med17 in chromatin (5) and 71 Lsm3 physical interactors, identified using the SGD (https://www.yeastgenome.org).

In a previous study (5), we used strains expressing Myc-tagged versions of the Med7 and Med17 Mediator subunits in pull-down experiments from chromatin extracts to identify proteins that specifically interact with the chromatin-bound form of Mediator. Among the 88 proteins we identified, 11 were subunits of the Pat1–Lsm complex. In order to complement and confirm these results, we here used a strain expressing TAP-tagged Lsm3 (21) to perform reciprocal experiments, i.e. that Lsm3 can pull down Mediator in chromatin extracts. An untagged strain was used as a negative control. In agreement with our previous results, we found that immunoprecipitation of Lsm3 can pull down Mediator in two independently isolated chromatin protein extracts (Figure 1A and Supplementary Figure S2A). Since Lsm3 is part of both the Lsm1–7 and Lsm2–8 complexes, we also performed similar co-immunoprecipitation experiments using strains expressing Flag-tagged Lsm1 and Flag-tagged Lsm8, respectively. The results show that both Lsm1 and Lsm8 can pull down Mediator (Med1 and Med4) isolated from chromatin (Supplementary Figure S2B).

Next, we used the YeastRGB open-source initiative (22) to study cellular localization of a set of Lsm and Mediator subunits (Figure 1B). Lsm1 accumulated in the cytoplasm, Lsm8 was exclusively nuclear, whereas Lsm3 localized to both the cytoplasm and nucleus, supporting the findings that Lsm1–7 forms a primarily cytoplasmic complex, while Lsm2–8 shows a preferred nuclear localization (14). We found that most core Mediator subunits were exclusively nuclear, while the kinase module subunit Cdk8 was detected both in the nucleus and in multiple cytoplasmic foci.

We also made a comparison of our previously reported chromatin-associated Mediator interactome data of 88 proteins (5) with 71 Lsm3 interacting proteins retrieved from the SGD. We identified a set of 14 common interactors that function at different stages of RNA processing, including splicing [Prp19 (23)], translation [Cdc33 (24)] and mRNA decay [the Lsm–PAT1 complex subunits Dcp1/2, EDC3 and Xrn1 (25,26)] (Figure 1C). Lsm3 and Prp19 have both been found in transiently interacting complexes formed during spliceosome assembly and activation (27,28). Prp19 accumulates at specific chromatin locations peaking ∼500 bp downstream of the 3′-splice sites of IC genes and participates in co-transcriptional splicing of ribosomal protein (RP) RNAs in yeast (29).

### Lsm3 is enriched at 3′-exons of IC–RP genes

**Figure 2.**
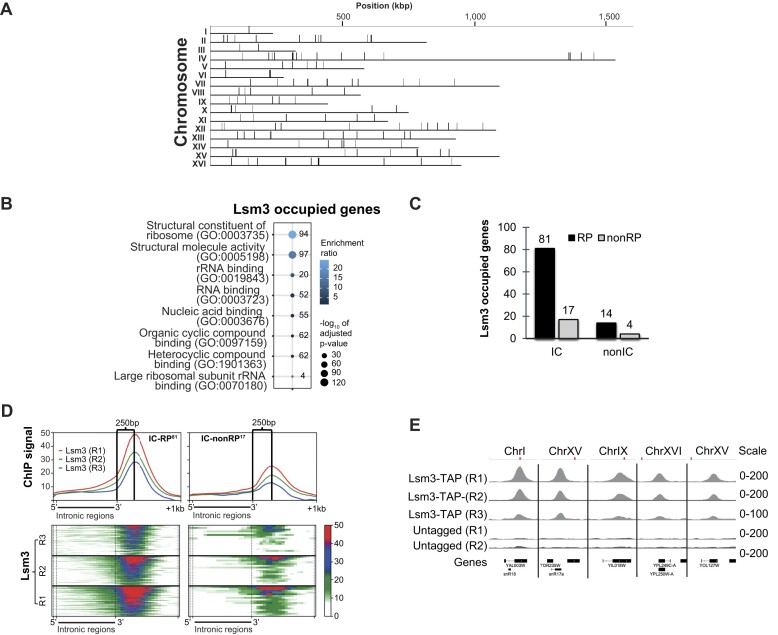
ChIP-seq analysis of Lsm3-TAP. (**A**) Overview of the locations of Lsm3 binding sites (vertical black lines) along the 16 yeast chromosomes. Top scale indicates chromosome size (kb). (**B**) Gene ontology (GO) analysis for the 116 Lsm3-occupied genes. Molecular functions with adjusted *P*-value ≤0.05 were extracted. Numbers to the right of circles indicate the number of Lsm3-occupied genes in each molecular function annotation. (**C**) Bar plot comparing the number of genes (top of each bar) of the four different classes of genes bound by Lsm3. IC, intron-containing genes; nonIC, genes that lack introns; RP, genes encoding ribosomal proteins; nonRP, genes encoding proteins other than ribosomal proteins. (**D**) Occupancy profiles (top) and heatmaps (bottom) of Lsm3 ChIP signals relative to the 3′-ends of the 81 IC–RP genes (left) and 17 IC–nonRP genes (right). Numbers on the top of each panel indicate the distance (bp) between the 3′-ends of introns and the peak summits. Replicate experiments are indicated as R1, R2 and R3. (**E**) IGV images from three independent Lsm3 ChIP-seq replicates compared to two independent replicate controls (untagged) at five representative IC genes. Red marks indicate the position of each gene relative to the chromosome starts and ends.

To determine global Lsm3 occupancy, we performed ChIP-seq assays using the *LSM3-TAP* strain and IgG-coupled Dynabeads. Three biological replicates of ChIP-seq assays were performed using an untagged strain as control (see Supplementary Figure S1B for comparisons between the biological replicates). We identified 127 Lsm3-occupied chromatin regions distributed over all 16 chromosomes (Figure 2A and Supplementary Table S1). Eleven Lsm3 peaks overlapping with 238 previously described HCRs (30) were removed from all subsequent analyses (Supplementary Figure S3A and Supplementary Table S2), even though most of these peaks neither overlapped extensively with the HCRs nor were detected in ChIP-seq assays using the untagged strain (Supplementary Figure S3B). The 116 remaining base coordinates were used to retrieve a list of 149 Lsm3-occupied genes. In 33 cases (66 genes/33 regions), 2 genes mapped to the same Lsm3-occupied region. These were of four types: convergent, parallel, embedded in intronic regions and having overlapping 3′-exons (Supplementary Table S2 and Supplementary Figure S3C and D). For the first three types, we included the gene that overlapped with the peak summit and omitted the gene that did not. For the fourth type, we removed genes encoding dubious ORFs according to the SGD to obtain a final set of 116 curated Lsm3-occupied genes (Supplementary Table S3).

GO analysis of the Lsm3-occupied genes showed highest enrichment of the molecular function categories ‘Structural constituent of ribosome’ and ‘Structural molecule activity’ (Figure 2B and Supplementary Table S4). Accordingly, we found that 81% of the Lsm3-occupied genes (94 of 116 genes, *P*-value = 4.6 × 10^−157^) were RP genes but also that 84% were IC genes (97 of 116 genes, *P*-value = 2.2 × 10^−109^) (Figure 2C). The 116 genes were further subdivided into 81 IC–RP, 17 IC–nonRP, 14 nonIC–RP and 4 nonIC–nonRP genes. Interestingly, several of the nonRP genes still encode proteins or RNAs connected to ribosome functions/assembly or translation. One IC–nonRP gene encodes ACT1 that functions in translational fidelity (31) and three other (*TEF4*, *NOG2* and *IMD4*) encode proteins involved in different aspects of ribosome function (32–34). These three genes each encompass one small nucleolar RNA (snoRNA) gene (*SNR38*, *SNR191* and *SNR54*, respectively) that is encoded in their introns. These snoRNAs are by themselves encoded by nonIC–nonRP genes and have been shown to play functional roles in translation (35,36). In addition, the IC–nonRP gene *SNR17B* encodes an snoRNA, which is a component of the small ribosome subunit processosome complex (37). Finally, we note that 9 of the 11 Lsm3-occupied genes that we removed due to their vicinity to HCRs were RP genes, and all 11 were IC genes.

**Figure 3.**
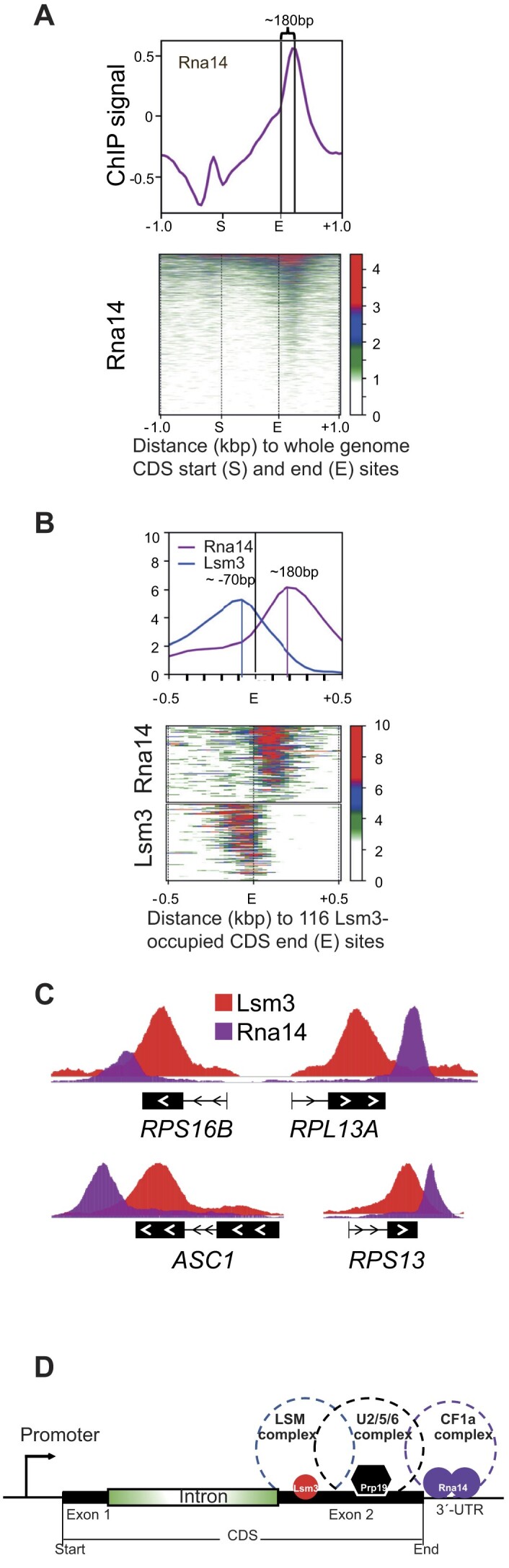
Comparison between Lsm3 and Rna14 binding at the 3′-ends of genes. (**A**) Global occupancy profile (top graph) and global heatmap (bottom graph) of Rna14-TAP ChIP signal relative to the CDSs (±1 kb). Distance (bp) between CDS end sites and peak summit is indicated on the top of the peak. CDSs (S to E) were scaled to 1 kb. (**B**) Occupancy profiles (upper graph) and heatmaps (bottom graph) of Lsm3-TAP and Rna14-TAP ChIP signals upstream and downstream of the CDS end sites for the 116 Lsm3-occupied genes. Distances (bp) between CDS end sites and peak summits are indicated on the top of each peak. (**C**) IGV images showing comparisons between Lsm3 and Rna14 ChIP-seq signals at representative examples of four typical IC–RP genes. (**D**) A model representing the mapped locations for the LSM decaysome (Lsm3), the U2/5/6 spliceosome (Prp19) and the CF1a 3′-end processing (Rna14) complexes at a model IC–RP gene.

Since the Lsm2–8 complex is involved in splicing (13), we made occupancy heatmap profiles to determine the location of Lsm3 peaks in the 81 IC–RP and 17 IC–nonRP genes (Supplementary Table S5 and Figure 2D and E). In both classes, Lsm3 occupied the last exons with a summit ∼250 bp downstream of their last intron 3′-ends. To correlate chromatin positions of the entire set of 116 Lsm3-occupied DNA regions to transcription termination, we performed ChIP-seq using a TAP-tagged Rna14 subunit of the pre-mRNA cleavage factor 1a complex (CF1a), which is involved in pre-mRNA 3′-end processing and contains several subunits that were previously identified by us as interacting with the chromatin-bound form of Mediator (5,38). Globally, Rna14 was enriched in the 3′-untranslated regions (UTRs) of 401 genes, at a mean distance ∼180 bp downstream of the 3′-ends of their CDSs (Figure 3A). Of these genes, 80 were commonly occupied by both Lsm3 and Rna14 (Figure 3B and Supplementary Table S6). By comparing binding of Lsm3 and Rna14 at the 116 Lsm3-occupied genes, we found that Lsm3 bound at a mean position around 70 bp upstream of the 3′-ends of the CDSs, ∼250 bp upstream of the position for Rna14 binding in their 3′-UTRs (Figure 3B and C).

The peak of Lsm3 occupancy at ∼250 bp downstream from the last intron in IC–RP genes partly overlaps with chromatin regions in the last exon of IC genes previously identified as enriched for binding of the Prp19 spliceosome subunit (29), which is important for co-transcriptional splicing of IC–RP genes and was previously identified by us as interacting with the chromatin-bound Mediator (5). Prp19 occupies the 3′-exons but further downstream (∼500 bp) of their 3′-splice sites relative to Lsm3. However, Prp19 still binds in their CDSs. In combination with our finding that Rna14 binds to the 3′-UTRs, and thus downstream of Prp19, these results suggest a possible model for a coordinated role between Lsm3, Prp19 and Rna14 in maturation of IC–RP transcripts (Figure 3D).

### Lsm3 occupancy at 3′ exons correlates with Mediator occupancy at promoter regions of IC–RP genes

Based on our earlier biochemical findings of interactions between Mediator and the Lsm complex in chromatin, we performed ChIP-seq experiments using affinity-purified antibodies specific for Med1 and Med15 in the wild-type strain *BY4742*. Genome-wide, we identified Mediator occupancy at 869 (Med1) and 648 (Med15) regions (Supplementary Figure S4A and Supplementary Table S7). The genomic regions (±500 bp from the peak starts and ends) at sites enriched >2-fold relative to the IgG control in each Mediator ChIP-seq were used to retrieve a list of 2171 Med1 binding and 1344 Med15 binding genes using the YeastMine database (Supplementary Table S8). Of these, 1300 sites were commonly occupied by both Med1 and Med15 (Supplementary Figure S4B). This list was manually curated as described in the ‘Materials and methods’ section to reach a final number of 725 protein encoding genes commonly occupied by both Med1 and Med15 (Supplementary Figure S4B and Supplementary Table S9). Heatmaps showed that Mediator predominantly occupies the promoter regions of these genes (Supplementary Figure S4C). However, both Med1 and, to a lesser extent, Med15 also occupied the CDSs of some genes (see genes at the top of each heatmap). For comparison and validation, we used score files from our previously published ChIP-seq data (5) for a set of TAP-tagged Mediator subunit strains representing each of the four Mediator modules, head (TAP-Med17), middle (TAP-Med14, TAP-Med19), tail (TAP-Med3, TAP-Med15) and kinase (TAP-CycC), to study their occupancy to the 725 genes identified here in ChIP-seq experiments using anti-Med1 and anti-Med15 antibodies (Supplementary Figure S4D). All TAP-tagged subunits showed enrichment in the promoter regions but also, to a lesser extent, in the CDSs of these genes.

**Figure 4.**
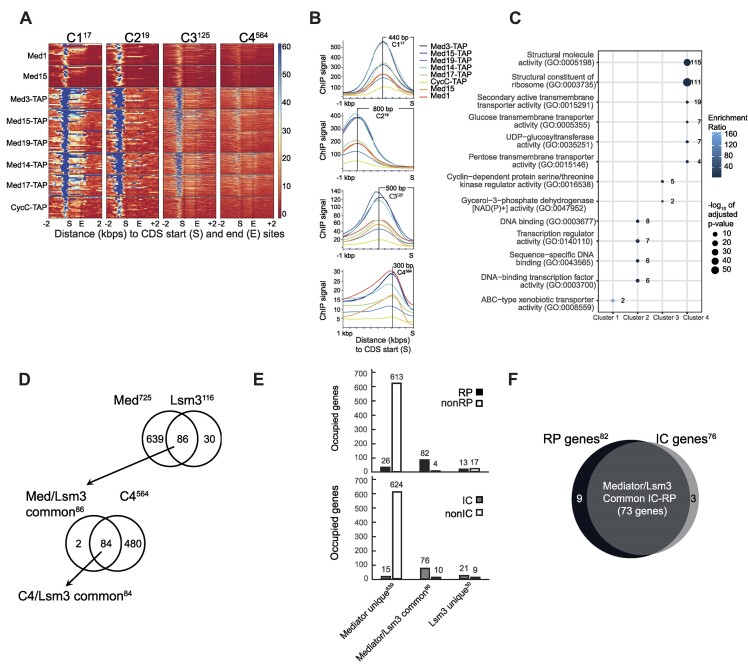
Mediator binds 200 bp upstream of transcription start site to 86 (74%) of the 116 Lsm3-occupied genes, of which 73 (85%) are IC–RP genes. (**A**) The 725 Mediator-bound genes were arranged into four clusters by *k*-means clustering. The ChIP signals (counts per million per 10-bp bin) of each peak are shown in the clustered heatmaps. Score files of ChIP-seq data from this study (Med1 and Med15) and previous study (5), using TAP-tagged Mediator subunits, were combined to compute matrices for the heatmaps. The numbers of genes in each cluster are indicated as superscript. (**B**) The profile plots of Mediator binding patterns at the promoter regions of clusters 1–4. Average ChIP-seq signals for each cluster and distances between peak summits and CDS start sites are plotted. (**C**) GO analysis for genes in each cluster. Molecular functions with adjusted *P*-value ≤0.05 were extracted. Numbers to the right of circles indicate the number of genes in each molecular function annotation. Colors of circles represent the enrichment ratio of cluster frequency relative to the genome frequency. Sizes of circles represent the adjusted *P*-values. (**D**) The upper Venn diagram (top) shows that Mediator (occupying 725 genes) and Lsm3 (occupying 116 genes) co-occupy 86 common genes. The lower Venn diagram shows that 84 of these 86 genes are found in cluster 4. (**E**) Bar plots showing the number of genes from each gene class (IC/nonIC or RP/nonRP) uniquely bound by Mediator, by both Mediator and Lsm3, or by Lsm3 uniquely. (**F**) Venn diagram representing the overlap of 73 genes between the 82 RP genes and the 76 IC genes that are co-occupied by Mediator and Lsm3. These 73 IC–RP genes that are co-occupied by Mediator and Lsm3 constitute 81% of all 90 IC–RP genes, 53% of all 138 RP genes and 19% of all IC genes identified in the yeast genome.

We combined our ChIP-seq data using Med1 and Med15 antibodies in wild-type cells with the data derived from TAP-tagged strains and arranged the 725 genes into four groups by *k*-means clustering (Figure 4A and Supplementary Table S10). To better describe the characteristics of each cluster, we plotted occupancy profiles in the promoter regions (Figure 4B) and found that the clusters differed with respect to their peak heights and distances from their peak summits to the start of their CDSs. GO analysis of genes in each cluster showed that they were enriched for distinct molecular functions (Figure 4C and Supplementary Table S11). Cluster 1 was enriched for genes encoding ABC-type xenobiotic transporters, cluster 2 for genes encoding proteins involved in GOs related to transcriptional regulation and cluster 3 for genes encoding proteins related to cyclin-dependent kinase regulatory and glycerol-3-phosphate dehydrogenase activities. Finally, cluster 4 was highly enriched for genes encoding proteins involved in ribosomal function and assembly, similarly to what we found for the 116 genes occupied by Lsm3 (Figure 2B). Interestingly, by comparing all 725 Mediator-occupied genes with the 116 Lsm3-occupied genes, we found an overlap of 86 genes, which corresponds to 74% of the Lsm3-occupied genes, and 84 of these genes were found in cluster 4 (Figure 4D). The remaining two Mediator/Lsm3 co-occupied genes (*RPS4A* and *ACT1*) were found in cluster 3. Based on our findings for the Lsm3-occupied genes (Figure 2C), we compared the enrichment of IC and RP genes between the 725 Mediator-occupied genes and the 116 Lsm3-occupied genes (Figure 4E). We found that the 86 common Mediator- and Lsm3-occupied genes were highly enriched for both RP (82 of 86 genes = 95%, *P*-value = 2.3 × 10^−148^) and IC (76 of 86 genes = 88%, *P*-value = 5.2 × 10^−89^) genes. In contrast, neither the uniquely Mediator-occupied nor the uniquely Lsm3-occupied genes were enriched for RP or IC genes. In total, we found that 73 of the 86 (85%) common Mediator- and Lsm3-ocupied genes were IC–RP genes (Figure 4F).

### IC–RP genes co-occupied by Mediator and Lsm3 show a second Mediator peak in their 3′-exon overlapping with the peak for Lsm3

**Figure 5.**
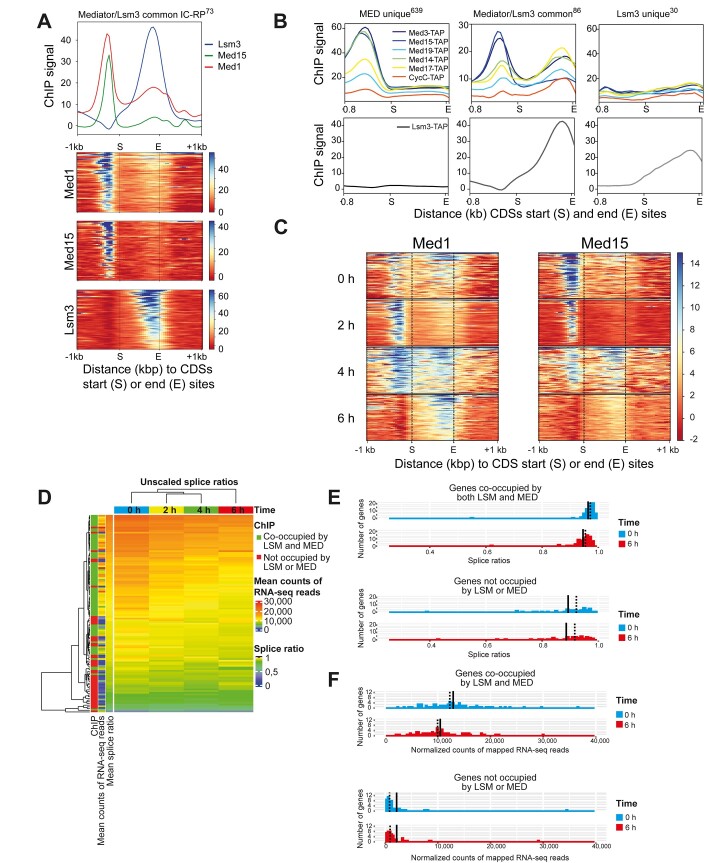
Mediator and Lsm3 show co-occupancy at 3′-exons of IC–RP genes in the late exponential growth phase. (**A**) Occupancy profiles and heatmaps of Med1, Med15 and Lsm3 ChIP-seq signals of the 73 commonly occupied IC–RP genes. Gene CDSs are scaled to 1 kb. (**B**) Occupancy profiles of TAP-tagged Mediator subunits (tail: Med3 and Med15; middle: Med19 and Med14; head: Med17; kinase: CycC) relative to the CDSs of the 639 genes uniquely occupied by Mediator (left panel), the 73 commonly occupied IC–RP genes (middle panel) and the 30 genes uniquely occupied by Lsm3 (right panel). Score files for TAP-tagged subunits were extracted from our previous ChIP-seq study (8). Gene CDSs are scaled to 1 kb. (**C**) Heatmaps of Med1 and Med15 ChIP-seq signals from different growth stages at the 73 commonly occupied IC–RP genes. Gene CDSs are scaled to 1 kb. (**D**) Heatmap with dendrograms for time points (on top) and genes (left) produced using Euclidean distance and complete linkage from the average of splice ratios at each time point for wild-type cells grown at 30°C. The left bars aligned with the heatmap represent the gene ChIP-binding characteristics, the average count of mapped RNA-seq reads for all time points and the average splice ratio for all time points. The names of the genes, from top to bottom, are listed in Supplementary Table S15 using the same color code as in the figure. (**E**) Distributions of the average splice ratios at the 0 and 6 h time points for IC genes co-occupied by Lsm3 and Mediator (upper) and IC genes without Lsm3 or Mediator binding (lower). Paired *t*-test *P*-values for the 6 h time points compared to the 0 h time points were 7.4e−12 (upper graph) and 0.088 (lower graph). (**F**) Distributions of the average normalized counts of mapped RNA-seq reads at the 0 and 6 h time points for IC genes co-occupied by Lsm3 and Mediator (upper) and IC genes without Lsm3 or Mediator occupancy (lower). Paired *t*-test *P*-values for the 6 h time points compared to the 0 h time points were 8.3e−21 [upper versus lower graph in panel (E)] and 0.95 [upper versus lower graph in panel (F)]. Solid and dashed lines in panels (E) and (F) represents mean and median values, respectively.

We next produced heatmaps and occupancy profiles to compare the location of Med1, Med15 and Lsm3 binding in the 73 co-occupied IC–RP genes (Figure 5A). We again observed a second Mediator peak at the 3′-ends of these genes coinciding with the Lsm3 peak, especially for the Med1 ChIP-seq (cf. Supplementary Figure S4C). For an independent comparison, we used our previously reported ChIP-seq data using TAP-tagged Mediator strains and compared occupancy of Mediator and Lsm3 to each of the 639 genes exclusively bound by Mediator, the 86 genes that are co-occupied by Mediator and Lsm3, and the 30 genes that are exclusively bound by Lsm3 (Figure 5B and Supplementary Figure S4D). For the Mediator unique genes, we only found Mediator occupancy in their promoter regions and no Lsm3 binding to any part of these genes (Figure 5B, left panels). For the Mediator/Lsm3 co-occupied genes, we found Mediator occupancy at both their promoters and 3′-ends, while Lsm3 showed strong occupancy uniquely to their 3′-ends at a position overlapping the second Mediator peak and no detectable Lsm3 at the promoters (Figure 5B, middle panels). Finally, Lsm3 showed occupancy only to the 3′-ends of the Lsm3 unique genes, but we detected no binding of Mediator to any part of this group of genes (Figure 5B, right panels). We noticed a relatively stronger occupancy of Lsm3 at the 86 Mediator/Lsm3 co-occupied genes relative to the 30 Lsm3 uniquely occupied genes suggesting that the Mediator and Lsm complexes might bind cooperatively to the co-occupied genes. The absence of Lsm3 at the strongest site of Mediator occupancy in the promoters further shows that the co-occupancy of Mediator and Lsm3 in the 3′-end of the genes is specific for that location.

### Mediator occupancy shifts from the promoters to the 3′-ends of IC–RP genes when cells approach the late logarithmic phase, and this transition correlates with reduced expression levels and reduced splicing of these genes

RP genes are expressed at their highest rates at mid-exponential growth but are downregulated in the late exponential phase when cells approach the diauxic shift due to glucose exhaustion (39). We compared Mediator binding to the 73 IC–RP genes at different time points during early to late exponential phases (Figure 5C; see Supplementary Table S12 for growth curves). During the early to early/mid-exponential phase (0 and 2 h), we detected significant occupancy of Mediator to the IC–RP gene promoters and less to their 3′-ends. We observed a marginally increased occupancy of Mediator at the 3′-ends at the 0 h time point compared to the 2 h time point, particularly noticeable in the Med1 experiment. We interpret this phenomenon as a lingering effect stemming from the recent dilution of these cells from overnight cultures. However, during the mid-exponential phase (4 h), Mediator showed near-equal occupancy to both their promoters and 3′-ends. Finally, during the late exponential phase (6 h), Mediator predominantly occupied the 3′-ends and showed no detectable promoter occupancy. As a control, we also analyzed the ChIP results from the IgG control experiments, but found no major differences in the background signals when comparing the 2 and 4 h time points (Supplementary Figure S4E). For a more detailed view, please refer to the IGV images illustrating comparisons between Lsm3, Med1 and Med15 occupancies at the 0, 2, 4 and 6 h time points for representative examples of four typical IC–RP genes (see Supplementary Figure S4F). In conclusion, our results indicate that the transition from exponential growth to lag phase correlates with a transition of Mediator from the promoters of IC–RP genes to the position of Lsm3 occupancy at their 3′-ends, a shift that might affect both mRNA levels and splicing efficiency.

We next used an unbiased RNA-seq-based approach to study the effects of Mediator transition on both mRNA levels and splicing efficiencies. Wild-type BY4742 cultures were grown to different densities as described for the ChIP-seq experiments, and samples for RNA-seq were harvested (see Supplementary Table S13 for growth curves). Analyses of the RNA-seq results identified 4733 genes that had at least 75 counts in all samples (six biological repeats for each time point). After filtering (see the ‘Materials and methods’ section), we obtained a list of 141 IC genes that could be used in comparisons to our ChIP-seq data (Supplementary Table S14). Out of these, 72 were genes that we detected as co-occupied by both Mediator and Lsm3 in our ChIP-seq, 45 were not occupied by either Mediator or Lsm3, 17 were uniquely occupied by Lsm3 and 7 were uniquely occupied by Mediator. For each IC RNA species, we estimated the fraction of spliced transcripts by calculating the relative abundance of RNA-seq mapped reads in whole genes compared to exons only. A scatter plot of the mRNA levels and splice ratios for each gene identified substantial differences between the 72 Mediator/Lsm3 co-occupied genes and the 45 genes that do not bind either Mediator or Lsm3 (Supplementary Figure S5A; see Supplementary Table S14 for specification of the genes that correspond to each number). Heatmap and cluster analyses of these two groups of genes (Figure 5D) clearly show that genes co-occupied by Mediator and Lsm3 (shown in green in the bar to the left) display higher and more homogeneous splice ratios as well as higher and more inhomogeneous expression levels compared to genes that bind neither Mediator nor Lsm3 (shown in red in the bar to the left; see Supplementary Table S15 for a list of the genes ordered from the top to the bottom in Figure 5D). These differences are more evident and quantified in histograms representing the expression levels (Supplementary Figure S5B) and splicing ratios (Supplementary Figure S5C) for all genes in each of the two groups. Finally, we used the RNA-seq data to study possible changes in expression levels and splice ratios for the co-occupied IC genes relative to the IC genes that bind neither Mediator nor Lsm3 at different growth states. Our results show that the Mediator and Lsm co-occupied IC genes showed significant reductions in both splice ratios (Figure 5E, upper panel) and expression levels (Figure 5F, upper panel) as cells go from early (0 h) to late (6 h) logarithmic growth. In contrast, IC genes that do not bind either Mediator or Lsm3 displayed no significant changes in either splice ratios (Figure 5E, lower panel) or expression levels (Figure 5F, lower panel). These differences are also visualized and quantified in box plots (Supplementary Figure S5D and E). Finally, we note that the co-occupied IC genes are spliced more efficiently and expressed at a much higher level in all growth phases than IC genes that do not bind either Mediator or Lsm3 (Figure 5E and F).

### Mediator is most likely interacting with the nuclear form of Lsm1–7

**Figure 6.**
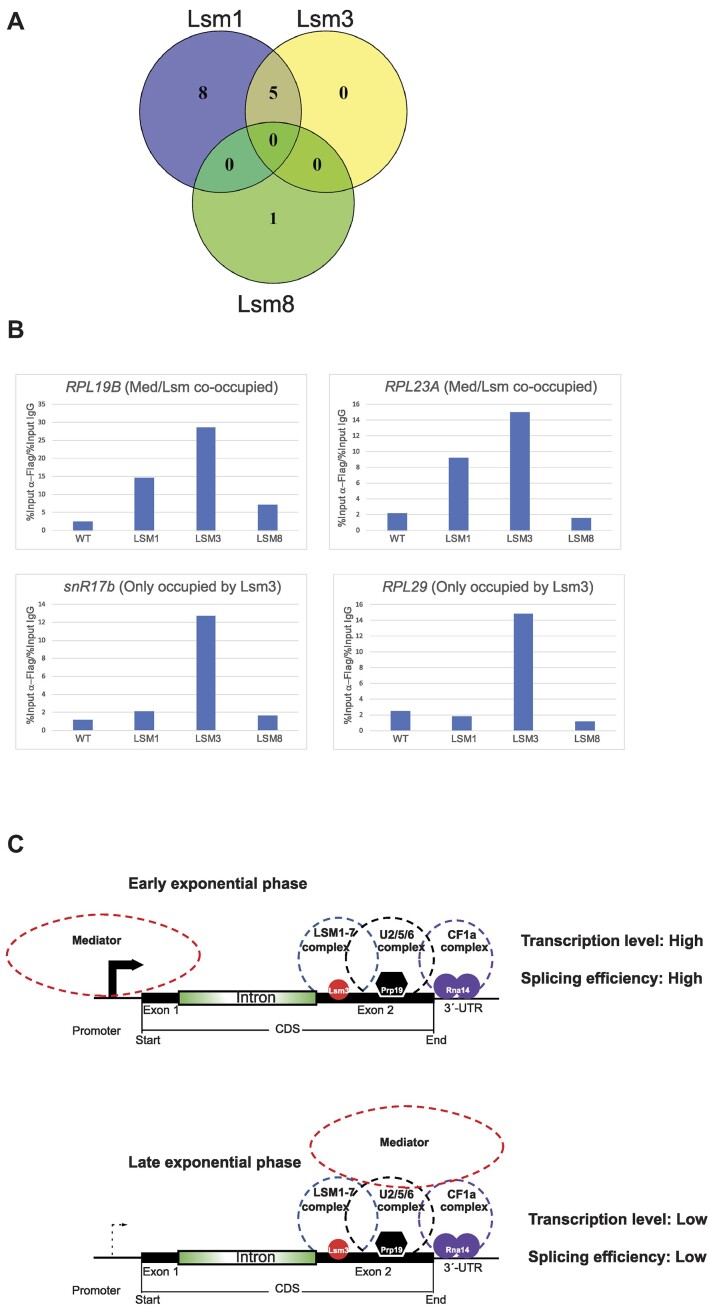
Interaction between Mediator and the Lsm1–7 complex at IC–RP genes. (**A**) Venn diagram illustrating the interactions between Lsm1, Lsm3 and Lsm8 subunits with Mediator subunits according to the SGD. (**B**) ChIP-qPCR experiments to assess the occupancy of each Lsm1-, Lsm3- and Lsm8-Flag tagged protein at two genes (*RPL19B* and *RPL23A*) that are co-occupied by Lsm3, Med1 and Med15 according to our ChIP-seq results, and at two genes (*snR17B* and *RPL29*) that are exclusively occupied by Lsm3 but not Med1 or Med15. The untagged wild-type strain served as the negative control. (**C**) A schematic model illustrating the interactions between Mediator and the Lsm, spliceosome and CF1a complexes, elucidating their roles in regulating IC–RP gene transcription and splicing in early and late exponential growth phases.

Most of our experiments presented thus far were conducted using the Lsm3 subunit, which is a component of both major forms of the Lsm complex, Lsm1–7 and Lsm2–8. Consequently, our ChIP-seq results cannot distinguish between these two forms. However, certain experiments suggest that Lsm1–7 is the more likely interaction candidate. First, we utilized the SGD to identify reported interactions between each of the Lsm1, Lsm3 and Lsm8 subunits and individual Mediator subunits. We found 13 reported genetic interactions between Lsm1 and various Mediator subunits (Med1, Med3/Pgd1, Med5/Nut1, Med8, Med9/Cse2, Med10/Nut2, Med12/Srb8, Med13/Ssn2, Med14/Rgr1, Med20/Srb2, Med31/Soh1, Cdk8/Ssn3 and CycC/Ssn8), 5 for Lsm3 (Med1, Med5/Nut1, Med8, Med9/Cse2 and Med20/Srb2) and only 1 (with Med6) for Lsm8 (Figure 6A). Notably, Lsm1 exhibits the highest count of reported interactions with Mediator, suggesting that the Lsm1–7 complex interacts with Mediator. Furthermore, there is a complete overlap between Lsm1 and Lsm3 for all five interactions documented for Lsm3. However, the sole interaction documented between Lsm8 and Mediator (with Med6) is not detected for either Lsm1 or Lsm3.

Subsequently, we conducted ChIP-qPCR experiments employing each of the Lsm1-, Lsm3- and Lsm8-Flag tagged strains. We utilized primers specific for two genes (*RPL19B* and *RPL23A*) that exhibit co-occupancy by Med1, Med15 and Lsm3 based on our ChIP-seq results, and primers specific for two genes (*snR17b* and *RPL29*) exclusively occupied by Lsm3, but not Med1 or Med15. Our findings revealed that both the Mediator/Lsm3 co-occupied genes were also occupied by Lsm1, but not by Lsm8 (see Figure 6B). Conversely, the genes exclusively occupied by Lsm3 were not occupied by either Lsm1 or Lsm8. Overall, our combined results, along with our previous report on interactions between Mediator and various proteins and protein complexes (including the Lsm1–7/Pat1 complex), support a model in which the Lsm1–7 complex interacts with Mediator at a majority of all IC–RP genes (Figure 6C).

## Discussion

Mediator was originally identified in budding yeast as a transcriptional co-activator required for integration of signals from promoter-bound transcriptional activators and repressors, and for transmission of these signals to the general Pol II preinitiation complex (1,40,41). More recently, Mediator has been linked to various other nuclear functions such as establishment of superenhancers (42), formation of topologically associating domains (chromosomal interaction domains in yeast) (5), transcription elongation (43), nucleosome displacement (44), splicing (45), termination (5) and mRNA export (46).

Our results presented here show that the yeast Lsm complex subunit Lsm3 and the Mediator tail and middle subunits Med15 and Med1 co-occupy positions in a specific set of IC ribosomal genes. In the early logarithmic growth phase, Mediator is located in the promoter regions of these genes, while Lsm3 occupies a region in the last exon, around 250 bp downstream of the intron–exon boundary. As cells exit the logarithmic growth phase, we detect a prominent transition of Mediator from the promoters of these genes to their 3′-exons where it becomes co-localized with the Lsm complex. This suggests a combinatorial, and functional, repressive effect on the expression levels of these genes by downregulating both their transcript levels and their splicing efficiencies. It is conceivable that the increased splice ratios observed for the Lsm–Mediator co-occupied genes could be influenced by their initially higher transcription levels. However, the prevailing literature suggests the opposite correlation: that high transcription rates are associated with decreased splicing efficiencies, and vice versa (47,48). Our findings reveal an unconventional pattern where Mediator transitioning from the promoter regions to the 3′-ends of IC–RP genes during the late exponential phase leads to a simultaneous reduction in both transcript levels and splicing efficiency. This observation deviates from the typical correlation between transcription rates and splicing efficiency and therefore suggests that it represents a unique feature for these genes in these conditions. Some previous evidence links transcription to splicing (49,50), and perturbations that lower the spliceosome assembly rate or increase transcription rates are known to cause a delay in splicing (47). A link between mRNA processing and transcription is also suggested by reports indicating crosstalk between metazoan Med23 and the splicing machinery regulator huRNP L (45), and by experiments showing that the *Arabidopsis thaliana* Med25 subunit couples alternative splicing of *JAZ* genes to fine-tuning of jasmonate signaling (51). It is widely recognized that Mediator plays a crucial role in modulating the phosphorylation status of the C-terminal domain of the largest Pol II subunit (1). This phosphorylation pattern governs key post-transcriptional processes such as 5′-capping, splicing and 3′-end processing (52–54). Therefore, it is conceivable that Mediator recruitment to a region downstream of the final intron of IC–RP genes could affect their splicing efficiency.

In line with our finding of an enrichment of Mediator tail and middle module subunits at the promoters of several RP genes, previous Chromatin endogenous cleavage sequencing (ChEC-seq) analysis of the Med8 head module subunit indicated robust enrichment at most RP gene promoters under normal growth conditions in either rich or minimal media (55). In contrast, early studies of Mediator occupancy at upstream activation sequences did not detect signals at promoters of highly expressed genes, including glycolytic genes and some RP genes (56,57). This was a surprising observation and was explained by variations in the association/dissociation dynamics of Mediator, specifically at RP genes (58). More recently, altered dynamics of Mediator tail occupancy at upstream sequences of RP genes have been described, which could explain the prominent Med15 occupancy at repressed and activated gene promoters during heat shock stress (59).

It is possible that Mediator has multiple effects in the maturation of RP mRNAs. We previously reported that the chromatin-bound form of Mediator interacts with spliceosome-associated subunits of the pre-mRNA cleavage factor CF1a (5). Our ChIP-seq experiments reported here using a strain expressing a TAP-tagged version of the CF1a subunit Rna14 combined with Prp19 ChIP-seq results from others suggest a model for the spatial relationship of Lsm to the spliceosome subunit Prp19 and the Rna14 subunit of the pre-mRNA cleavage factor CF1a at the 3′-end of these genes (Figure 3D).

An alternative possibility is that the overlap between Med1 and Lsm3 at 3′-exons of IC–RP genes is due to a gene looping-mediated transcription termination/reinitiation mechanism. In budding yeast, gene looping of many genes, including RP genes, was shown to accompany intron-mediated enhancement of transcription, which was also shown to depend on proper splicing (60,61). They found that the IC–RP *ASC1* exhibited a 25-fold decrease in expression when comparing full-length ASC1 to intron-deleted ASC1, while transcription run on analysis that measures the level of nascent transcripts still attached to the elongating polymerase on the template indicated significant accumulation of Pol II at the 3′-end of genes (60). However, the complete absence of Lsm3 from the Mediator-bound promoter regions (Figure 5A and B) argues against looping being the main reason for the co-occupancy of Mediator and Lsm3 at the 3′-exons of the IC–RP genes.

Altogether, our results suggest a model where specific Mediator subunits and Lsm3 may cooperate to regulate the expression of RP genes in an intron-dependent and splicing-mediated manner. It is possible that this regulation also involves interaction of Mediator with the spliceosome and CF1a (Figure 6C). In the future, it will be intriguing to discern the specific Mediator, Lsm, spliceosome and CF1a subunits directly implicated in this mechanism. Additionally, investigating the cause-and-effect relationship between Mediator/Lsm3 occupancy and the expression levels of genes, such as the removal of introns from typically co-occupied genes, holds promise for deeper insights.

## Supplementary Material

gkae266_Supplemental_Files

## Data Availability

The RNA-seq and ChIP-seq data underlying this article are available in the Gene Expression Omnibus database under accession code GSE254761.

## Notes

Present address: Wael R. Abdel-Fattah, Department of Biomedical and Clinical Sciences, Linköping University, SE-581 83 Linköping, Sweden.
