# Correction to “Growth-regulated co-occupancy of Mediator and Lsm3 at intronic ribosomal protein genes”

**DOI:** 10.1093/nar/gkae1258

**Published:** 2024-12-19

This is a correction to: Wael R Abdel-Fattah, Mattias Carlsson, Guo-Zhen Hu, Ajeet Singh, Alexander Vergara, Rameen Aslam, Hans Ronne, Stefan Björklund, Growth-regulated co-occupancy of Mediator and Lsm3 at intronic ribosomal protein genes, Nucleic Acids Research, Volume 52, Issue 11, 24 June 2024, Pages 6220–6233, https://doi.org/10.1093/nar/gkae266

The GEO accession number in the data availability statement was missing a digit and has now been updated to “GSE254761”.

This change does not affect the results, discussion and conclusions presented in the article. The published article has been updated.

