# Supplementary material for: Growth-regulated co-occupancy of Mediator and Lsm3 at intronic ribosomal protein genes
Source: Nucleic Acids Res. 2024 Apr 13;52(11):6220–33. doi: 10.1093/nar/gkae266 (PMC11194063; doi:10.1093/nar/gkae266)
Supplement: gkae266_Supplemental_Files [file gkae266_supplemental_files.zip › Supplementary Information__Abdel-Fattah_revised.pdf]

Growth regulated co-occupancy of Mediator and Lsm3 at intronic ribosomal protein genes.

Wael R. Abdel-Fattah[1#], Mattias Carlsson[2], Guo-Zhen Hu[2], Ajeet Singh[1], Alexander Vergara[3], Rameen Aslam[1], Hans Ronne[2] & Stefan Björklund[1*]

[1]Department of Medical Biochemistry and Biophysics, Umeå university, SE-901 87, Umeå, Sweden

[2]Department of Forest Mycology and Plant Pathology, Swedish University of Agricultural Sciences, Box 7026, SE-750 07 Uppsala, Sweden

[3]Department of Plant Physiology, Umeå university, SE-901 87 Umeå, Sweden

[#]Present address, Department of Biomedical and Clinical Sciences Linköping University SE-581 83 Linköping, Sweden

Supplementary data

Supplementary data contains captions for Supplementary Figures S1-S5, captions for Supplementary Tables S1-S15, and Supplementary Figures S1-S5.

Supplementary data figure legends

Supplementary Figure S1. Linear correlation profiles between the three biological replicates of the whole genome. (A) IgG (Control), **(B)** Lsm3-TAP, **(C)** Med1, **(D)** Med15. (1 kbp bin size).

Supplementary Figure S2. Lsm3 and Lsm8 interaction with Mediator in chromatin. (A) Co-immunoprecipitation of Med1 and Lsm3-TAP from chromatin. Two independently purified chromatin extracts (I1 and I2 (See Figure 1A for extract 1) were isolated from a strain expressing Lsm3-TAP, and immunoprecipitated using IgA magnetic beads. Chromatin extracts from an untagged strain (E_U) was used a negative control. Beads were washed with buffer A and proteins bound to the beads were eluted using 1 x SDS sample buffer. Proteins from the input (I2, 10 times diluted), eluate (E2) and wash (W2) were resolved on 4-15% SDS-PAGE, transferred to a PVDF membrane and blotted using a-Protein A (lower blot) or a-Med1 (upper blot) antibodies followed by incubation in Clean-blotTM IP (HRP) detection reagent (Thermo Scientific). **(B)** Similar experiments as in Figure 1A and Supplementary Figure S2A, but using chromatin extracts isolated from strains expressing 3xFlag-tagged Lsm1 and 3xFlag-tagged Lsm8, respectively. Proteins from the input (I, 10 times diluted), eluate from the tagged strains (E_T), wash (W), and eluate from the untagged strains (E_U) extract from an untagged strain were resolved on 4-15% SDS-PAGE, transferred to PVDF membranes and blotted using α-Flag (upper blot), α-Med1 (middle blot) and $_a$-Med4 antibodies (lower blot). Quantifications of bands in **(A)** and **(B)** were conducted using the ImageJ software and normalized to the input (set to 1).

Supplementary Figure S3. ChIP-seq analysis of Lsm3 occupied regions. (**A**) Upset plot for the intersection between the 116 regions occupied by Lsm3 and the 238 hyper-ChIPable regions (HCRs). (**B**) Integrative genomics viewer (IGV) images showing comparisons of Lsm3 ChIP-seq signals relative to positions of HCRs. (**C**) IGV images representing each of the four examples where two genes mapped to the same Lsm3-occupied regions. Arrows indicate genes that were omitted from (red) or included in (green) the list of Lsm3 occupied genes. (**D**) Pie chart representing the four different types of omitted genes and the final set of 116 Lsm3 occupied genes. R1, R2 and R3 refer to the three biological replicates.

Supplementary Figure S4. Analysis of yeast Mediator complex occupancies in chromatin by ChIP-seq. (**A**) Bar plot showing the number of Mediator peaks with different ranges of fold enrichment (FE). Data represents MACS2 peaks of Med1 (869 peaks) and Med15 (648 peaks) from ChiP-seq analyses. (**B**) Workflow for the definition of significant Mediator-occupied protein-coding genes. 869 Med1 and 648 Med15 peaks showing ≥2 folds change in occupancy above control were used to make two lists of genes (2171 for Med1, and 1344 for Med15) where any part of their CDSs maps to DNA regions ±500 bps of the peak start/end sites. Of the 1301 genes commonly occupied by Med1 and Med15, 725 represent protein coding genes that were not removed due to the presence of overlapping HCRs, being dubious ORFs, ncRNA genes, or ARSs/centromeres/transposable elements, being occupied by broad peaks or having peaks located over or downstream of their CDSs. (**C**) Heat maps of Med1 and Med15 ChIP signals relative to the CDS (±1 kb) of the 725 Mediator occupied genes. (**D**) Heatmaps of Mediator subunits ChIP signals relative to the CDS (±1 kb) of the Mediator occupied genes. Score files for the TAP-tagged Mediator subunits were extracted from our previously published study (5)[4]. CDSs of genes were scaled to 1 kbp. **(E)** Heatmaps of IgG (Control), Med1 and Med15 ChIP-seq signals from the 2 h and 4 h time points at the 73 commonly occupied IC-RP genes. Gene CDSs are scaled to 1kb. **(F)** IGV images showing comparisons between Lsm3, Med1, and Med15 ChIP-seq signals at the 0h, 2h, 4h, and 6h timepoints for representative examples of four typical IC-RP genes.

Supplementary Figure S5. Genes co-occupied by Mediator and Lsm3 both display higher splice ratios and higher expression relative to genes that bind neither Mediator nor Lsm3 in logarithmically growing cells but show significant reductions of both as cells are in late logarithmic phase. (**A**) Scatter plot with average splice ratios on y-axis and average normalized mapped RNA-seq reads on x-axis for IC genes co-occupied by LSM and MED (green) and IC genes not occupied by LSM or MED (red). The numbers in the scatter plot represent individual genes as described in Supplementary Table S14. (**B**) Density distributions of the average normalized counts of mapped RNA-seq reads for IC genes not occupied by LSM or MED (upper), and IC genes co-occupied by LSM and MED (lower). (**C**) Density distributions of the average splice ratios for IC genes not occupied by LSM or MED (upper), and IC genes co-occupied by LSM and MED (lower). (**D**) Box plots of the difference in splice ratio in Wt cells between the 6 h and 0 h time points for IC genes not occupied by LSM or MED (left) and IC genes co-occupied by LSM and MED (right). Mean zero one-sample t-test p-values for the two groups were 0.088 and 7.4e-12, respectively, and the t-test p-value for

intergroup comparison was 0.013. (**E**) Box plots of the difference in normalized counts of mapped RNA-seq reads in Wt cells between 6 h and 0 h time points for IC genes not occupied by LSM or MED (left) and IC genes co-occupied by LSM and MED (right). Mean zero one-sample t-test p-values for the two groups were 0.095 and 8.3e-21, respectively, and the t-test p-value for intergroup comparison was 5.5e-19.

Supplementary Table legends

Supplementary Table S1. Data for the enriched DNA regions of three independent replicates of LSM3-TAP ChIP-seq experiments.

Supplementary Table S2. Lists of HCRs, Lsm3 occupied regions in each replicate, Lsm regions that overlap with HCRs and the 33 omitted genes. Overlapping regions are in bold style.

Supplementary Table S3. List of the 116 genes occupied by Lsm3.

Supplementary Table S4. Gene ontology (GO) analysis for molecular function of the 116 Lsm3 occupied genes. GO analyses was performed at https://www.yeastgenome.org/goTermFinder using a p-value <0.01.

Supplementary Table S5. Coordinates of coding DNA sequences (CDS) and intronic sequences of genes occupied by Lsm3.

Supplementary Table S6. Coordinates of coding DNA sequences (CDS) for genes occupied by Rna14 globally and of 80 genes co-occupied by Rna14 and Lsm3.

Supplementary Table S7. Peak coordinates for chromosomal regions occupied by Med1 or Med15 with ≥2-fold enrichment above background control using IgG.

Supplementary Table S8. Identified genes located ±500 bp relative to the start and end of each peak enriched >2-fold relative to control in the Med1 and Med15 ChIP-seq experiments using the YeastMine database. Genes in bold type are enriched in both Med1 and Med15.

Supplementary Table S9. Curated list of genes commonly occupied by Med1 and Med15.

Supplementary Table S10. Mediator occupied genes arranged in four groups by k-means clustering.

Supplementary Table S11. Gene ontology (GO) analysis for molecular function of the Mediator bound genes in each cluster. GO analyses was performed at https://www.yeastgenome.org/goTermFinder using a p-value <0.05.

Supplementary Table S12. Growth curves for ChIP-seq experiments. Single colonies of Saccharomyces cerevisiae BY4742 cells (Wt) were cultivated and harvested as described in Materials and methods. Mean OD600 measurements and standard deviations (SD) for two independent experiments are shown in linear (left) and logarithmic (right) scales.

Supplementary Table S13. Growth curves for RNA-seq experiments. Single colonies of Saccharomyces cerevisiae BY4742 cells (Wt) were cultivated and harvested as described in Materials and methods. Mean OD600 measurements and standard deviations (SD) for six independent experiments are shown in linear (left) and logarithmic (right) scales.

Supplementary Table S14. IC genes sorted by Lsm3 and Mediator binding. Of the 141 filtered IC genes from the RNA-seq assays, the 45 genes in the No Binding column were not occupied by neither Mediator nor Lsm3 in the ChIP-seq, the 72 genes in the Lsm3 and Mediator column were detected as co-occupied by both Mediator and Lsm3, the 17 genes in the Lsm3 column were uniquely occupied by Lsm3 and the 7 genes in the Mediator column were uniquely occupied by Mediator.

Supplementary Table S15. List of the order of genes in Figure 5D from top to bottom. The genes are color coded in the same way as in Figure 5D. Green color represents genes co-occupied by LSM and MED. Red color represents genes not occupied by LSM or MED.

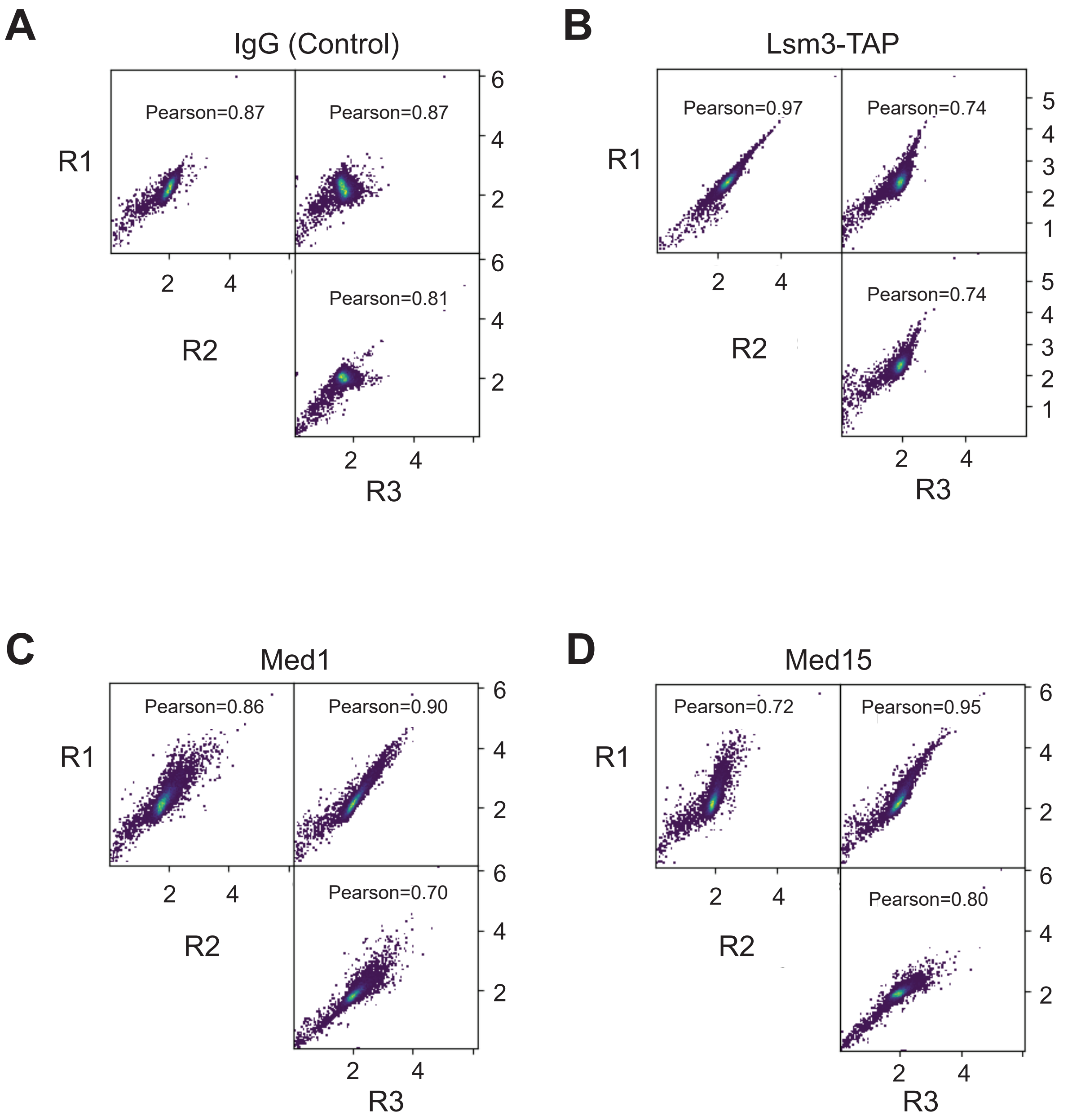

Supplementary Figure S1. Linear correlation profiles between the three biological replicates of the whole genome. **(A)** IgG (Control), (B) Lsm3-TAP, (C) Med1, (D) Med15. **(**1 kbp bin size).

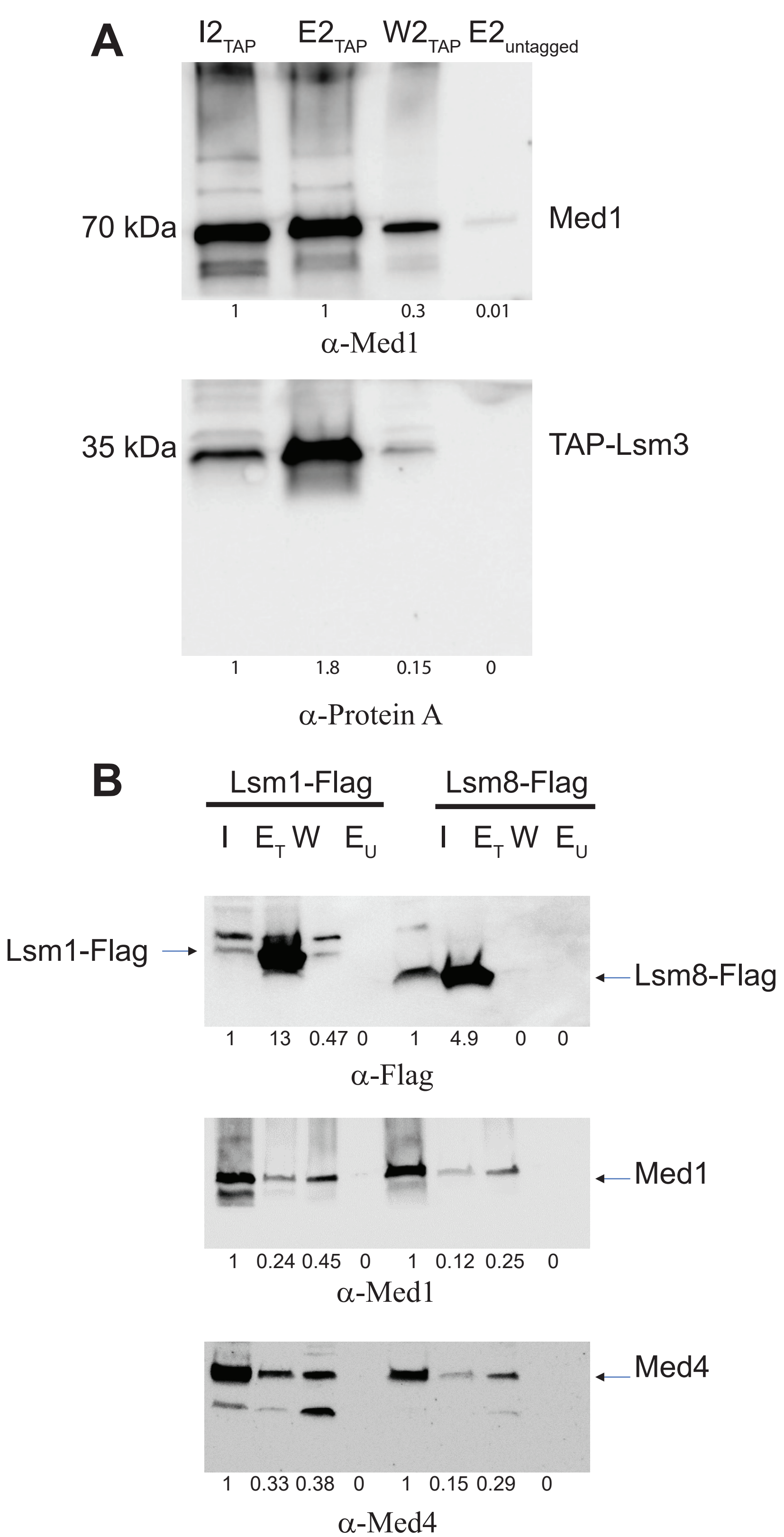

Supplementary Figure S2. Lsm3 and Lsm8 interaction with Mediator in chromatin. (A) Co-immunoprecipitation of Med1 and Lsm3-TAP from chromatin. Two independently purified chromatin extracts (I_1 and I_2 (See Figure 1A for extract 1) were isolated from a strain expressing Lsm3-TAP, and immunoprecipitated using IgA magnetic beads. Chromatin extracts from an untagged strain (E_U) was used a negative control. Beads were washed with buffer A and proteins bound to the beads were eluted using 1 x SDS sample buffer. Proteins from the input (I_2, 10 times diluted), eluate (E_2) and wash (W_2) were resolved on 4-15% SDS-PAGE, transferred to a PVDF membrane and blotted using α-Protein A (lower blot) or α-Med1 (upper blot) antibodies followed by incubation in Clean-blotTM IP (HRP) detection reagent (Thermo Scientific). **(B)** Similar experiments as in Figure 1A and Supplementary Figure S2A, but using chromatin extracts isolated from strains expressing 3xFlag-tagged Lsm1 and 3xFlag-tagged Lsm8, respectively. Proteins from the input (I, 10 times diluted), eluate from the tagged strains (E_T), wash (W), and eluate from the untagged strains (E_U) extract from an untagged strain were resolved on 4-15% SDS-PAGE, transferred to PVDF membranes and blotted using α-Flag (upper blot), α-Med1 (middle blot) and α-Med4 antibodies (lower blot). Quantifications of bands in **(A)** and **(B)** were conducted using the ImageJ software and normalized to the input (set to 1).

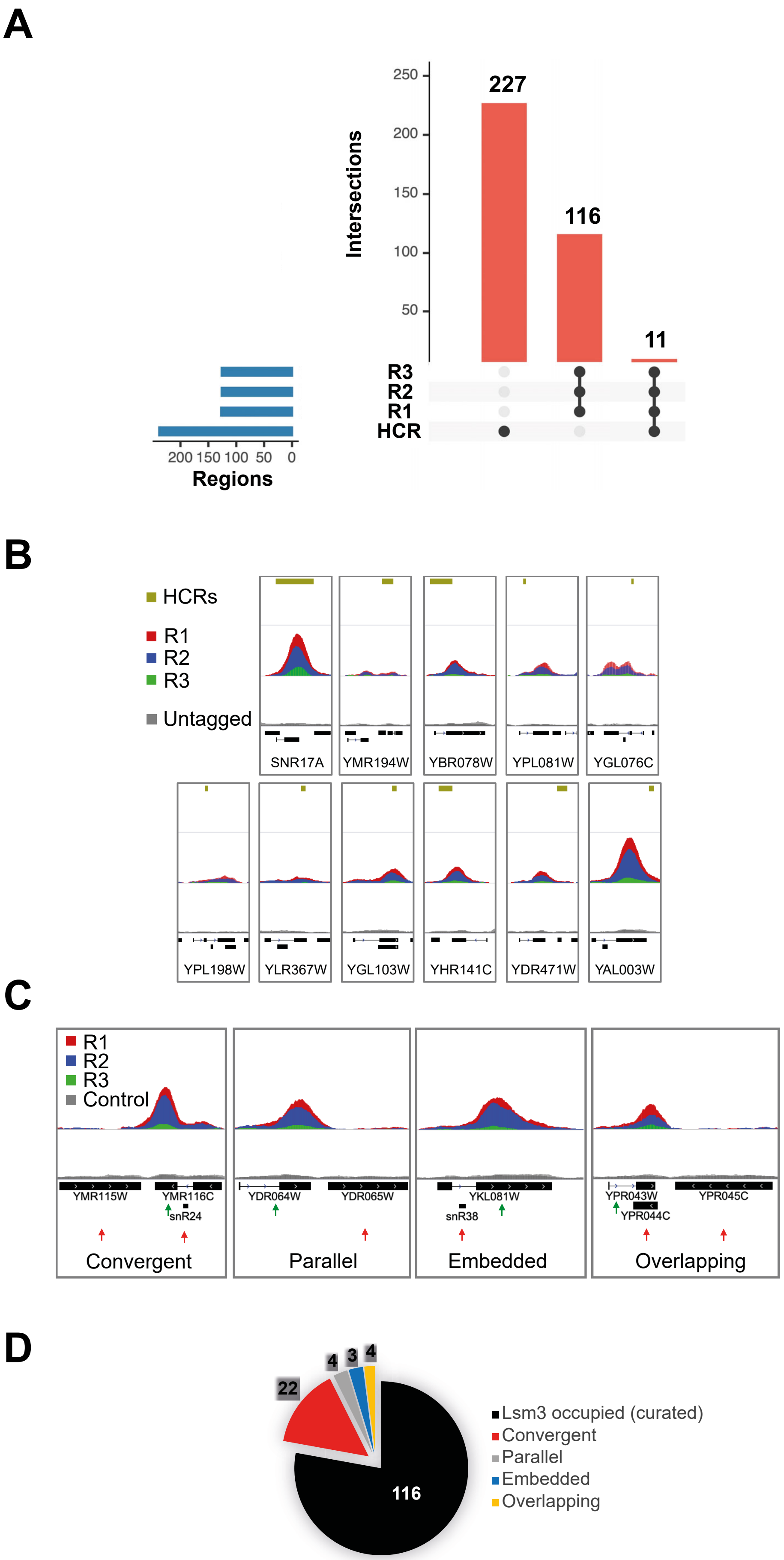

Supplementary Figure S3. ChIP-seq analysis of Lsm3 occupied regions. (A) Upset plot for the intersection between the 116 regions occupied by Lsm3 and the 238 hyper-ChIPable regions (HCRs). **(B)** Integrative genomics viewer (IGV) images showing comparisons of Lsm3 ChIP-seq signals relative to positions of HCRs. **(C)** IGV images representing each of the four examples where two genes mapped to the same Lsm3-occupied regions. Arrows indicate genes that were omitted from (red) or included in (green) the list of Lsm3 occupied gene list. **(D)** Pie chart representing the four different types of omitted genes and the final set of 116 Lsm3 occupied genes.

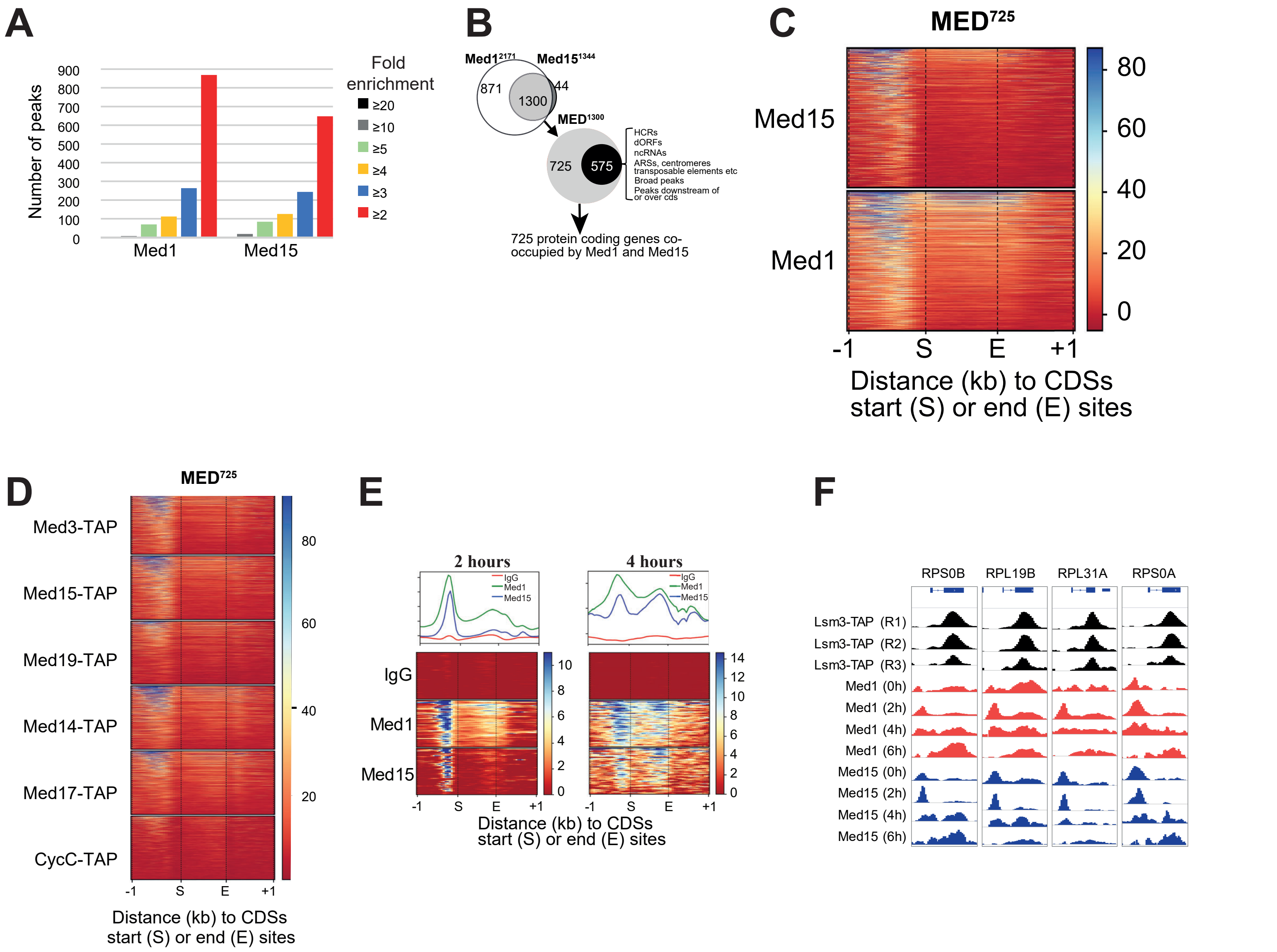

Supplementary Figure S4. Analysis of yeast Mediator complex occupancies in chromatin by ChIP-seq. (A) Bar plot showing the number of Mediator peaks with different ranges of fold enrichment (FE). Data represents MACS2 peaks of Med1 (869 peaks) and Med15 (648 peaks) from ChiP-seq analyses. **(B)** Workflow for the definition of significant Mediator-occupied protein-coding genes. 869 Med1 and 648 Med15 peaks showing ≥2 folds change in occupancy above control were used to make two lists of genes (2171 for Med1, and 1344 for Med15) where any part of their CDSs maps to DNA regions ±500 bps of the peak start/end sites. Of the 1300 genes commonly occupied by Med1 and Med15, 725 represent protein coding genes that were not removed due to the presence of overlapping HCRs, being dubious ORFs, ncRNA genes, or ARSs/centromeres/ transposable elements, being occupied by broad peaks or having peaks located over or downstream of their CDSs. **(C)** Heat maps of Med1 and Med15 ChIP signals relative to the CDS (±1 kb) of the 725 Mediator occupied genes. **(D)** Heatmaps of Mediator subunits ChIP signals relative to the CDS (±1 kb) of the Mediator occupied genes. Score files for the TAP-tagged Mediator subunits were extracted from our previously published study [4]. CDSs of genes were scaled to 1 kbp. **(E)** Heatmaps of IgG (Control), Med1 and Med15 ChIP-seq signals from the 2 h and 4 h time points at the 73 commonly occupied IC-RP genes. Gene CDSs are scaled to 1kb. **(F)** IGV images showing comparisons between Lsm3, Med1, and Med15 ChIP-seq signals at the 0h, 2h, 4h, and 6h timepoints for representative examples of four typical IC-RP genes.

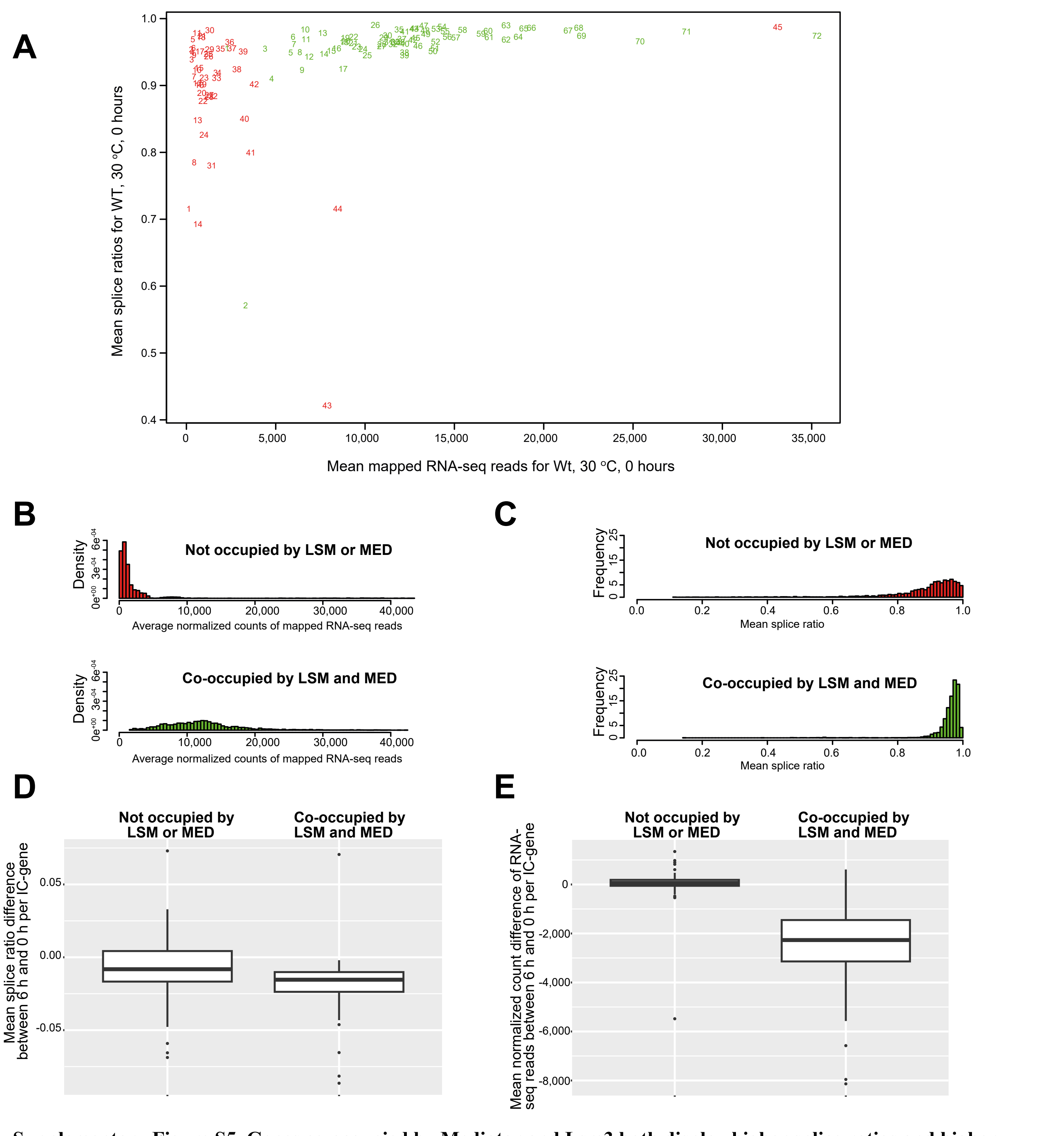

Supplementary Figure S5. Genes co-occupied by Mediator and Lsm3 both display higher splice ratios and higher expression relative to genes that bind neither Mediator nor Lsm3 in logarithmically growing cells but show significant reductions of both as cells approach stationary phase. (**A**) Scatter plot with average splice ratios on y-axis and average normalized mapped RNA-seq reads on x-axis for IC genes co-occupied by LSM and MED (green) and IC genes not occupied by LSM or MED (red). The numbers in the scatter plot represent individual genes as described in Supplementary Table S14. (**B**) Density distributions of the average normalized counts of mapped RNA-seq reads for IC genes not occupied by LSM or MED (upper), and IC genes co-occupied by LSM and MED (lower). (**C**) Density distributions of the average splice ratios for IC genes not occupied by LSM or MED (upper), and IC genes co-occupied by LSM and MED (lower). (**D**) Box plots of the difference in splice ratio in Wt cells between the 6 h and 0 h time points for IC genes not occupied by LSM or MED (left) and IC genes co-occupied by LSM and MED (right). Mean zero one-sample t-test p-values for the two groups were 0.088 and $7.4e^{-12}$, respectively, and the t-test p-value for intergroup comparison was 0.013. (**E**) Box plots of the difference in normalized counts of mapped RNA-seq reads in Wt cells between 6 h and 0 h time points for IC genes not occupied by LSM or MED (left) and IC genes co-occupied by LSM and MED (right). Mean zero one-sample t-test p-values for the two groups were 0.095 and $8.3e^{-21}$, respectively, and the t-test p-value for intergroup comparison was $5.5e^{-19}$.
